# Epidemiological profile of pain and non-steroid anti-inflammatory drug use in collegiate athletes in the United States

**DOI:** 10.1186/s12891-020-03581-y

**Published:** 2020-08-19

**Authors:** S. Christopher, B. A. Tadlock, B. J. Veroneau, C. Harnish, N. K. P. Perera, A. M. Knab, S. Vallabhajosula, G. S. Bullock

**Affiliations:** 1 Department of Physical Therapy Education, Elon University, Elon, NC USA; 2 Department of Exercise Science, Mary Baldwin College, Staunton, VA USA; 3 Kinesiology Department, Queens University of Charlotte, Charlotte, NC USA; 4 Centre for Sport, Exercise and Osteoarthritis Research Versus Arthritis, University of Oxford, Oxford, UK; 5 Nuffield Department of Orthopaedics, Rheumatology, and Musculoskeletal Sciences, University of Oxford, B4495, Oxford, OX3 7LD UK; 6 Unit of Physiotherapy, Department of Health, Medicine and Caring Sciences (HMV), Linköping University, Linköping, Sweden; 7 School of Allied Health, Human Services and Sport, Latrobe University, Melbourne, Victoria Australia; 8 Department of Orthopaedic Surgery, Wake Forest School of Medicine, Winston-Salem, North Carolina USA

**Keywords:** Sleep, Female athletes, Sports medicine, Health literacy, Athlete health, Injury prevention, Elite sport

## Abstract

**Background:**

Although athletic endeavours are associated with a high amount of physical stress and injury, the prevalence of pain is underreported in the sports medicine literature with only a few studies reporting pain on collegiate athletes or exploring sex difference of pain. Impact of pain on athlete availability, training and performance can be mitigated when key epidemiological information is used to inform adequate pain management strategies. This study aims to 1) provide an epidemiological profile of self-reported pain experienced by the National Collegiate Athletic Association (NCAA) athletes by sex during the first half of the 2019 season, 2) describe their self-reported non-steroidal anti-inflammatory drug (NSAID) use.

**Methods:**

Online survey was completed by athletes at three NCAA institutions from 1 August to 30 September 2019. Descriptive statistics were used to describe player demographic data, self-reported pain and self-reported NSAID use. Pain incidence proportion were calculated.

**Results:**

Two hundred thirty female athletes and 83 male athletes completed the survey. Self-reported pain incidence proportion for female athletes was 45.0 (95% CI 41.5–48.5) vs 34.9 (95% CI 29.4–40.4) for male athletes. Majority of the athletes did not report pain (55% female vs 62% male) during the first half of the 2019 season. Female athletes reported pain in their back (35%), knee (26%), and ankle/foot (23%) whilst male athletes reported pain in their knee (35%), back (28%), and shoulder (24%). Of all athletes, 28% female vs 20% male athletes reported currently taking NSAIDs. Of athletes that reported pain, 46% female vs 38% male athletes currently took NSAIDs. 70% female vs 61% male athletes self-purchased NSAIDs, and 40% female vs 55% male athletes consumed alcohol.

**Conclusions:**

Half of female athletes and one in three male athletes reported pain. Most commonly back, knee and foot/ankle pain and knee, back and shoulder pain was reported in female and male athletes respectively. One in four female athletes and one in five male athletes use NSAIDs for pain or prophylactic purpose. Majority self-purchase these medications indicating need for health literacy interventions to mitigate potential adverse effects.

## Background

The National Collegiate Athletic Association (NCAA) is a United States and Canadian based non-profit organisation that regulates and organises sports for over 480,000 student athletes in more than 1200 colleges and universities. The NCAA programs have three tiers, namely division one, division two, and division three athletic programs [1]. The NCAA competition is the highest level of competition for collegiate athletes with many NCAA athletes on athletic scholarship, potentially contributing to increased pressure to play [2]. Collegiate athletes, like many competitive athletes, invest substantial amount of time and effort to meet the physical demands of their sports. As a results, their bodies are under considerable amount of strain and pain [3]. Pain can be a precursor to the development of an overuse injury, over training, or associated with illness, and can decrease performance, training, and overall athlete availability [4–6].

Pain is multidimensional, incorporating neurological, biomechanical, and psychological constructs [7]. While pain is felt by both male and females, there are differences in pain response between genders [8, 9]. Previous research suggests females have a greater response to painful stimuli and overall pain reporting, with females more susceptible to chronic pain conditions, more sensitive to pain threshold and tolerance, and more readily report pain compared with males [8, 9].Although athletic endeavours are associated with a high amount of physical stress and injury [10], athletes may underreport pain due to distinct athletic factors such as team and coach pressure [11], significance of upcoming games [12], and psychological factors [13]. Thus, the prevalence of pain may be underreported in the sports medicine literature with only a few studies reporting pain on collegiate athletes or exploring sex difference of pain [8, 9, 14]. Impact of pain on athlete availability, training and performance can be mitigated when key epidemiological information is used to inform adequate pain management strategies [15].

Various methods are used by athletes to minimise the impact of pain during training and competition [16]. One of the most common pain management strategies is the use of Non-Steroidal Anti-Inflammatory Drugs (NSAID), a class of medications that reduces pain, decrease inflammation, and fever [17, 18]. Side effects depend on the specific NSAID, but the most common side-effects include an increased risk of gastrointestinal upsets, ulcers and bleeds, bronchospasm, bruising, raised liver enzyme, and kidney disease [18, 19]. Some NSAIDs (except aspirin) can increase the risk of heart attack and stroke, potentially life-threatening even in healthy people [20]. Further, if NSAIDs are used without medical guidance, drug interactions with other medicines could cause unwanted effects [18, 19]. To reduce risk of side-effects, NSAIDs must be used cautiously and occasionally rather than daily, at the lowest effective dose possible for the shortest time [18, 21]. Since NSAIDs are easy to access and affordable, athletes of all backgrounds frequently use it without any medical advice and has been seen to be used the highest at the high school or professional level [14, 18]. Given the potential poor health literacy among athlete population, prolonged use of NSAIDs, particularly prophylactic misuse is concerning [6, 18, 22]. For example, athletes were unable to differentiate between competition and training related soreness, and injury related pain, potentially leading to indiscriminate pain management strategies [6, 22]. Also, athletes are known to consume high amount of alcohol [23], taking high doses of NSAIDs (male athletes consumed 8% more NSAIDs compared to the general population) [24] and for more than a few days at a time (10% of male athletes take NSAIDs prior to each competition) [18]. Regular use of NSAIDS can increase the risk of gastrointestinal bleeding in individuals who consumed alcohol [22]. Owing to these known risk factors and prophylactic misuse of NSAIDs without appropriate medical advice, athletes are a NSAIDs high-risk group for developing serious complications [6, 18, 22]. Also, NSAID consumption might be different between sexes, for example female athlete report more pain, but data on NSAID use is not available [8]. General lack of data from a well-defined athlete population hinder development of evidence-based health literacy interventions as well as inform medical professional to deliver appropriately targeted care.

This study aims to 1) provide an epidemiological profile of self-reported pain experienced by NCAA athletes by sex during the first half of the 2019 season, 2) and describe their self-reported NSAID use.

## Methods

**Fig. 1:**
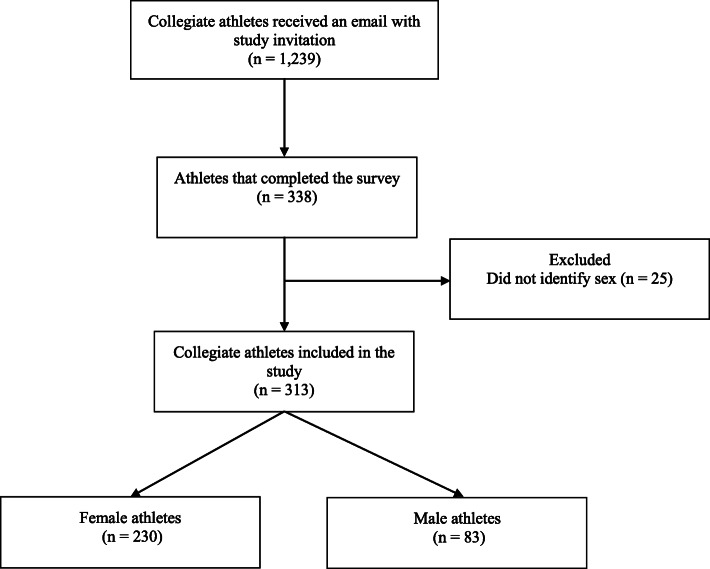
Study Participant Flow Chart

This cross-sectional survey was part of a larger collegiate health and well-being study on: (1) athlete general health; (2) lifestyle and academics; (3) pain, injury, and surgery and (4) well-being. Survey was adopted from the cricket health and well-being study [25] and pilot tested by three researchers (BT, VB, GB), three current collegiate athletes and a collegiate athletic trainer. Following ethics approval from the Elon University Institutional Review Board (ID: 20–026), a link to the online survey and the plain language information statement was disseminated by the sports medicine staff at three NCAA institutions (Elon University, Queens University of Charlotte, and Mary Baldwin College) to 1239 NCAA athletes via email. The online survey was live from 1 August to 30 September 2019 (8-weeks), inclusive via the Qualtrics software (Qualtrics, Provo, UT) and took average of 12 min to complete. To reduce participant burden, athletes could save their responses and return to complete the survey at their leisure. Two email reminders were sent at weeks two and six and verbal reminders were given by coaches during week four. Inclusion criteria was 1) an NCAA athlete who is enrolled at one of the three participating institutions, and 2) listed on the official roster for their sport. Study participant recruitment is illustrated in Fig. 1. The survey included 19 questions relating to player demographic characteristics, sports participation, pain, and NSAID use (Additional file 1).

Main outcome measures were current self-reported pain due to illness and injury reported using the Oslo Sports Traumatic Research Centre for Overuse Injury Questionnaire (OSTRC) where 0 = no pain, 8 = mild pain,, 17 = moderate pain and 25 = severe pain [4]. Pain scores were then dichotomised into no pain (score of 0) and presence of pain (score of > 0) for analysis. If athletes reported pain, they were asked to list affected joints (Additional file 1). Athletes NSAID use was the second main outcome measure and assessed using the question, “*Are you currently taking any Non-Steroidal Anti-Inflammatory Drug at this time for any reason for your sport?*^*26*^*”* (Additional file 1). In order to make meaningful sport comparisons, sports were categorised into collision sport (American football, rugby, and lacrosse), field and court sport (basketball, football, tennis, and volleyball), bat-and-ball and ball-and-stick sport (baseball and softball), and individuals sports (cross country, dance, golf, swimming, track and field, and triathlon).

Descriptive statistics were used to describe player demographic data, self-reported pain and self-reported NSAID use. Pain incidence proportion, with 95% confidence intervals (95% CI) for all athletes and by sex was calculated using the following formula [26].

$$ \mathrm{Pain}\ \mathrm{incidence}\ \mathrm{proportion}=\frac{\#\mathrm{of}\ \mathrm{athletes}\ \mathrm{reported}\ \mathrm{pain}}{\#\mathrm{of}\ \mathrm{all}\ \mathrm{athletes}\ }\ \mathrm{x}\ 100 $$

The relationship between self-reported NSAID use and self-reported current pain was explored using unadjusted and adjusted logistic regression with 95% CI’s. All logistic regression models were adjusted for NCAA division, Body Mass Index (BMI), alcohol use, surgical history, injury history, and number of hours sleeping. All confounders were included in the full model. All statistical analyses were performed in R version 3.5.1. When possible, categories were broadened in a meaningful way to ensure ≥5 cases to protect athletes’ privacy. All data cells corresponding to counts of < 5 were replaced by an asterisk (*) in the presentation of results in order to ensure confidentiality.

Missing data were analysed through counts, percentages, and visualisation through the R package naniar. Missing data was determined to be low, and that variables were missing at random except for where athletes receive NSAIDs (Additional file 2). Due to this, participant data was split for descriptive analyses into missing and not missing groups for the NSAID questions. Mann-Whitney U and chi-square tests were performed between groups for age, BMI, NCAA division, surgical history, and injury history and no differences were observed between groups. It was concluded that missing data for the question where athletes receive NSAIDs was missing not completely at random (MNAR). Further, due to these findings, logistic regression models only analysed NSAID use or no NSAID use, and all other data relating to NSAID use were analysed descriptively.

## Results

A total of 1239 male and female athletes received the survey. The survey response rate was 26% where 230 female athletes (Division 1: 39%, Division 2: 33%, and Division 3: 28%) and 83 male athletes (Division 1: 52%, Division 2: 19%, Division 3: 29%) completed the survey (Table 1).

**Table 1:** Participant descriptive characteristics and health-related and study habit statistics

	Female Total (*n* = 230)	Female Division 1 (*n* = 89)	Female Division 2 (*n* = 77)	Female Division 3 (*n* = 64)	Male Total (*n* = 83)
Age (years)	19.0 (18–20)	19.0 (18–20)	19.0 (18.5–19.5)	19.0 (18–20)	20.0 (19.5–20.5)
Height (m)	1.71 (.09)	1.73 (.09)	1.69 (.09)	1.69 (.08)	1.87 (.08)
Weight (kg)	67.6 (11.9)	68.1 (12.2)	66.8 (12.1)	67.9 (11.4)	87.9 (21.5)
Academic Rank					
Freshman	85 (37%)	29 (33%)	26 (34%)	30 (47%)	42 (51%)
Sophomore	70 (30%)	25 (27%)	26 (34%)	19 (30%)	16 (19%)
Junior	47 (21%)	21 (24%)	14 (18%)	12 (18%)	11 (13%)
Senior	28 (12%)	14 (16%)	11 (14%)	3 (5%)	12 (14%)
Sport Played					
Collision	*(*%)	*(*%)	*(*%)	*(*%)	13 (15%)
Field and Court	97 (42%)	35 (39%)	36 (47%)	26 (41%)	27 (33%)
Bat, Ball, and Stick	54 (23%)	29 (33%)	8 (10%)	17 (27%)	26 (31%)
Individual	71 (31%)	22 (25%)	29 (38%)	20 (31%)	15 (18%)
NA	6 (3%)	*(*%)	*(*%)	*(*%)	*(*%)
Seasons Played at the Collegiate Level	2 (0–4)	2 (0–4)	2 (0–4)	2 (0–4)	2 (0–4)
Ethnicity					
White	170 (74%)	59 (66%)	61 (79%)	50 (78%)	67 (81%)
Black or African American	28 (12%)	16 (18%)	6 (8%)	5 (8%)	7 (8%)
Hispanic or Latino	12 (5%)	5 (7%)	*(*%)	*(*%)	*(*%)
Native American or Native Alaskan	*(*%)	*(*%)	*(*%)	*(*%)	*(*%)
Do not wish To answer	18 (8%)	8 (9%)	6 (8%)	*(*%)	5 (6%)
Sleep (hours)					
< 7	72 (31%)	21 (24%)	22 (29%)	29 (45.5%)	16 (19%)
7–8	122 (53%)	51 (57%)	42 (46%)	29 (45.5%)	59 (71%)
9+	16 (7%)	7 (8%)	7 (9%)	* (*%)	* (*%)
NA	20 (9%)	10 (11%)	6 (6%)	*(*%)	* (*%)
Alcohol Consumption in last 30 days					
Yes	104 (32%)	54 (39%)	25 (26%)	25 (27%)	31 (37%)
No	184 (56%)	66 (48%)	60 (62%)	58 (64%)	48 (58%)
NA	38 (12%)	18 (13%)	12 (12%)	8 (9%)	* (*%)

*NA* Not answered

*(*%) are used when counts are below 5 in order to protect participant identification

Data are reported as mean (standard deviation), mean (range-range), or count (percent)

Self-reported pain incidence proportion for female athletes was 45.0 (95% CI: 41.5–48.5) vs 34.9 (95% CI: 29.4–40.4) for male athletes. Sport specific self-reported pain incidence proportion for collision sport played by female 100.0 (95% CI: 100.0, 100.0) vs male 69.2 (95% CI: 56.4–82.0)); field and court sports played by female 44.3 (95% CI: 39.3–49.4) vs male 44.4 (95% CI: 34.9–54.0); bat-and-ball and ball-and-stick sports played by female 31.5 (95% CI: 25.2–37.8) vs male 19.2 (95% CI: 11.5–7.0); individual sports played by female 40.8 (95% CI 35.0–46.7) vs males 20.0 (95% CI: 9.7–30.3). Division 1 pain incidence proportion was 34.4 (95% CI: 29.4–39.5) for females and 51.2 (95% CI: 43.5–58.8) for males. Division 2 pain incidence proportion was 33.8 (95% CI: 28.4–39.2) for females and 6.25 (95% CI: 0.2–12.3) for males. Division 3 pain incidence proportion was 53.1 (95% CI: 46.9–59.4) for females and 25.0 (95% CI: 16.2–33.8) for males.

Majority of the athletes did not report pain (55% female vs 62% male) during the first half of the 2019 season. Of the athletes who self-reported pain, most athletes reported mild pain (76% female vs 59% male) (Table 2).

**Table 2:** Comparison of athletes with and without self-reported pain

Female athletes									Male athletes	
	All Divisions		Division 1		Division 2		Division 3		All Divisions	
	Current Pain (*n* = 91)	No Current Pain (*n* = 111)	Current Pain (*n* = 31)	No Current Pain (*n* = 44)	Current Pain (*n* = 26)	No Current Pain (*n* = 43)	Current Pain (*n* = 34)	No Current Pain (*n* = 24)	Current Pain (n = 29)	No Current Pain (*n* = 47)
Alcohol use in past 30 days										
Yes	36 (40%)	35 (32%)	14 (45%)	15 (34%)	9 (25%)	11 (26%)	13 (38%)	9 (28%)	16 (55%)	13 (28%)
No	55 (60%)	76 (68%)	17 (55%)	29 (66%)	17 (65%)	32 (74%)	21 (62%)	15 (63%)	13 (45%)	34 72%)
Hours of Sleep										
< 7 h	39 (43%)	28 (25%)	13 (42%)	5 (11%)	9 (35%)	12 (28%)	17 (50%)	11 (46%)	5 (17%)	10 (21%)
7–8 h	45 (49%)	75 (68%)	15 (48%)	34 (77%)	14 (54%)	28 (65%)	16 (47%)	12 (50%)	23 (79%)	34 (72%)
> 9 h	7 (8%)	8 (7%)	*(*%)	*(*%)	*(*%)	*(*%)	*(%)	*(*%)	* (*%)	* (*%)
History of Surgery										
Yes	20 (22%)	12 (11%)	5 (16%)	*(*%)	9 (25%)	5 (12%)	6 (18%)	*(*%)	8 (28%)	6 (13%)
No	71 (79%)	99 (89%)	26 (84%)	41 (93%)	17 (65%)	38 (88%)	28 (82%)	20 (83%)	21 (72%)	41 (87%)
History of injury led to > 4 weeks’ time loss from sport										
Yes	12 (13%)	46 (41%)	23 (74%)	19 (43%)	20 (77%)	19 (44%)	18 (53%)	8 (33%)	24 (83%)	17 (36%)
No	99 (87%)	65 (59%)	8 (26%)	25 (57%)	6 (335)	24 (56%)	16 (47%)	16 (66%)	5 (17%)	30 (64%)
Sport Played										
Collision	*(*%)	* (*%)	*(*)	*(*%)	*(*%)	*(*%)	*(*%)	*(*%)	9 (31%)	*(*%)
Field and Court	43 (47%)	46 (41%)	16 (52%)	16 (36%)	13 (50%)	20 (47%)	14 (41%)	10 (42%)	12 (41%)	14 (29%)
Bat, Ball, and Stick	17 (19%)	29 (26%)	7 (23%)	17 (39%)	*(*%)	*(*%)	6 (18%)	9 (28%)	5 (17%)	21 (45%)
Individual	29 (32%)	35 (23%)	7 (23%)	11 (25%)	8 (31%)	19 (44%)	14 (41%)	5 (21%)	3 (10%)	9 (19%)
Current NSAID use										
Yes	42 (46%)	23 (21%)	16 (52%)	7 (16%)	8 (31%)	9 (21%)	18 (53%)	7 (29%)	11 (38%)	6 (15%)
No	45 (49%)	87 (78%)	13 (42%)	37 (84%)	18 (69%)	33 (77%)	14 (41%)	13 (54%)	17 (59%)	41 (87%)
No Answer	* (*%)	* (*%)	*(*%)	*(*%)	*(*%)	*(*%)	*(*%)	*(*%)	* (*%)	* (*%)

Count discrepancies between female and male participants is due to missing data

Data are reported as count (percent)

*(*%) are used when counts are below 5 in order to protect participant identification

*NSAID* Non-Steroidal Anti-Inflammatory Drug

**Fig. 2:**
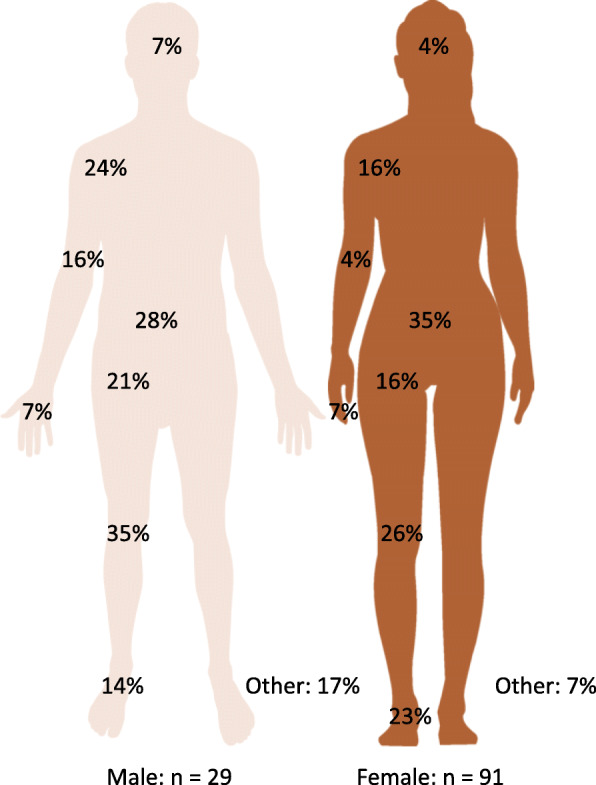
Anatomical location of the self-reported joint pain in female and male collegiate athletes during the first half of the 2019 season

Female athletes reported the greatest prevalence of back pain (35% female vs 28% male), compared to male athletes who reported the greatest prevalence of knee pain (26% female vs 35% male). Female athletes reported a high prevalence of pain in their ankle/foot (23% female vs 14% male), in comparison to males who reported a high prevalence of shoulder pain (16% female vs 24% male; Fig. 2).

Of all athletes, 28% female vs 20% male athletes reported to currently taking NSAIDs. Of the athletes that reported pain, 46% female vs 38% male athlete currently consume NSAIDs. Also, 21% female vs 15% male athletes that did not report pain were taking NSAIDs (Table 2). 41% female vs 63% male athletes reported never consuming NSAIDs during the off-season and 30% females vs 47% males never consumed NSAIDs during the season. Female athletes that currently consume NSAIDs, 36% had consumed alcohol in the past 30 days vs 37% of male athletes (Table 3).

**Table 3:** Female and male athlete non-steroid anti-inflammatory drug use

	Female Total (*n* = 197)	Male Total (*n* = 75)
NSAID Use In Season		
3-7 days/week	22 (11%)	5 (7%)
1-2 days/week	43 (22%)	13 (17%)
1-3times/month	73 (37%)	22 (29%)
Never	59 (30%)	35 (47%)
NSAID Use Off Season		
3-7 days/week	8 (4%)	0
1-2 days/week	23 (12%)	7 (9%)
1-3times/month	85 (43%)	21 (28%)
Never	81 (41%)	47 (63%)
Receiving NSAIDs		
Self-Purchased	98 (70%)	25 (61%)
Coaches	0	0
Parents	23 (16%)	7 (17%)
Healthcare Professionals	15 (11%)	9 (22%)
Teammates	*(*%)	0
Other	*(*%)	0
NSAID Dosage		
2 pills	106 (83%)	29 (74%)
4 pills	10 (8%)	10 (16%)
6+ pills	11 (9%)	0

Data are reported as count (percent)

*(*%) are used when counts are below 5 in order to protect participant identification

Differences in count data are due to missing data

*NSAID* Non-Steroidal Anti-Inflammatory Drug

Female athletes that reported current pain had greater odds of currently taking NSAIDs (Unadjusted: 3.53 (95% CI: 1.91–6.66), *p* < 0.0001; Adjusted: 2.97 (95% CI: 1.51–5.97), *p* = 0.002; Table 4)**.** Male athletes that reported current pain had greater unadjusted odds of using NSAIDs (Unadjusted: 4.42 (95% CI: 1.45–14.7), *p* = 0.009). However, there was no adjusted relationship (Adjusted: 3.36 (95% CI: 0.80–16.8), *p* = 0.176; Table 5.

**Table 4:** Multivariable logistic regression analysis investigating the relationship between pain and NSAID use in female athletes

	Unadjusted Odds Ratio (95% CI)	Adjusted Odds Ratio^a^ (95%CI)
Current NSAID use and pain (OSTRC) (Female)	3.53 (1.91, 6.66), *P* = < 0.0001	2.97 (1.51, 5.97), *P* = 0.002
Division 1		Reference
Division 2		0.99 (0.46, 2.13), *P* = 0.988
Division 3		2.09 (0.93, 4.78), *P* = 0.078
BMI		0.98 (0.89, 1.08), *P* = 0.701
Alcohol in Last 30 Days		0.80 (0.41, 1.58), *P* = 0.521
Surgery History		1.27 (0.51, 3.25), *P* = 0.614
Injury History		3.03 (1.56, 6.04), *P* = 0.001
< 7 Hours of Sleep		Reference
7–8 Hours of Sleep		0.54 (0.27, 1.08), *P* = 0.081
> 9 Hours of Sleep		1.17 (0.32, 4.24), *P* = 0.811

^a^ Estimates were adjusted for NCAA division, BMI, if alcohol was consumed within the last 30 days, history of surgery, history of injury, and hours of sleep

**Table 5:** Multivariable logistic regression analysis investigating the relationship between pain and NSAID use in male athletes

	Unadjusted Odds Ratio (95% CI)	Adjusted Odds Ratio^a^ (95%CI)
Current NSAID use and pain (OSTRC) (Male)	4.42 (1.45, 14.7), P = < 0.0001	3.36 (0.80, 16.8), *P* = 0.112
Division 1		Reference
Division 2		0.07 (0.003, 6.26), *P* = 0.038
Division 3		0.88 (0.19, 4.05), *P* = 0.869
BMI		1.14 (0.98, 1.35), *P* = 0.077
Alcohol in Last 30 Days		0.37 (0.08, 1.51), *P* = 0.174
Surgery History		0.70 (0.11, 34.23), *P* = 0.699
Injury History		15.8 (3.53, 96.9), *P* < 0.001
< 7 Hours of Sleep		Reference
7–8 Hours of Sleep		1.39 (0.03, 47.3), *P* = 0.093
> 9 Hours of Sleep		0.37 (0.08, 1.51), *P* = 0.174

^a^ Estimates were adjusted for NCAA division, BMI, if alcohol was consumed within the last 30 days, history of surgery, history of injury, and hours of sleep

## Discussion

One of the most important findings of the present study was that self-reported pain incidence proportion was higher in female athletes and almost half of female athletes compared to one in three male athletes reported pain. We found that one in four female athletes and one in five male athletes were currently using NSAIDs and almost one in two female and male athletes with current pain consumed alcohol. Females with current had greater odds of consuming NSAIDS, after adjusting for confounders.

Female athletes reported greater pain incidence in comparison to males. Females have a greater response to painful stimuli and overall pain reporting [8, 9], and are more susceptible to chronic pain conditions, sensitive to pain threshold and tolerance, and readily report pain compared with males [8, 9]. Despite playing similar sports and competing at the same standard of play, females reported greater pain incidence; however, females were overrepresented in this sample. It should also be noted that these athlete participated different sports, and therefore the physical demands may not be the same. These sport participation discrepancies may alter pain incidence and reporting [27, 28]. Nevertheless, these results potentially suggest that female collegiate athletes may have different potential pain responses to similar sporting activities, and that clinicians should consider these pain response differences between sexes when monitoring and evaluating collegiate athletes.

Sex differences were observed for the anatomical location of joint pain. Female athletes reported pain in their back, knee, and ankle/foot and males reported pain in their knee, back, and shoulder. Interestingly, while females usually have a higher risk of knee pain [29], these results demonstrated that the greatest pain prevalence was to back in female athletes. Further, prevalence of back pain was greater in female athletes than males. The disparity in back pain reporting has been attributed to hip muscular imbalances due to anatomical and strength differences between sexes [30]. Low back pain has also been associated with lower extremity pain and injury in female athletes, such as to the knee or ankle/foot [31], further corroborating our results. The prevalence of lower extremity pain, specifically to the knee and foot/ankle may be due to the increased risk of lower extremity injury in females [29]. These anatomical pain discrepancies between females and males should be considered when evaluating and interpreting collegiate athlete pain.

Female athletes that reported current pain had greater odds of currently taking NSAIDs compared to female athletes without pain. NSAIDs are the foremost popular medication consumed to mask musculoskeletal pain [5]. Musculoskeletal pain can diminish performance through decreased force production, endurance, or the inability to perform specific athletic skills [32]. Due to the high demand and competitive nature of collegiate athletics, collegiate athletes are inclined to try to maintain performance, despite pain and possible injury [3]. The statistical models in our study observed that division was not a significant factor in determining NSAID use. Further, athletes potentially use NSAIDs in order to reduce the debilitating effects of pain in pursuit of remaining competitive and available [18]. Within this study, 28% of female athletes were currently taking NSAIDs, which is comparable to previous literature [33]. Interestingly, 43% reported self-purchasing NSAIDs, 16% received NSAIDs from family members, while only 12% received NSAIDs from athletic trainers or team physicians, and it is unknown if these athletes sought medical advice prior to NSAID consumption. Only one study has previously investigated where collegiate athletes receive their NSAIDs, finding higher proportions of NSAID purchase by the individual athletes (59%) and family members (22%) [33]. However, this study only investigated male college athletes at one institution [33]. As NSAIDs are relatively cheap and can be obtained over the counter, there is a low barrier to obtaining these drugs. Athletes have shown a general lack of knowledge concerning the ability to identify the differences between athletic soreness and injury, and the implications and side effects of NSAID use [22]. These educational and discriminatory issues can have important harmful health consequences, possibly contributing to increased injury or drug side effects [22]. Collegiate athletic departments, no matter the NCAA division level, and sports medicine clinicians need to educate athletes and their families on proper NSAID use, and identify barriers to monitoring NSAID consumption in athletes.

Male collegiate athletes reported an 8% lower use of NSAIDs in comparison to female athletes. Further, while there was a greater unadjusted odds of NSAID use, after adjusting for confounders, there was no relationship. As stated previously, females are more at risk to have pain, which may be due to hormonal differences between sexes [34]. These potential biological differences may explain these NSAID use discrepancies. However, while these results are disparate in comparison to the female college athletes, with the significant decrease in the male sample (Females: 75%, Males: 25% of all respondents) and reported wide confidence intervals, these results should be interpreted with caution.

### Limitations

#### As with all studies, there were limitations

Pain is multidimensional and the epidemiological profile of pain can change throughout the year. Collegiate athletes train and compete in sport throughout the year and our data only provide a snapshot at a single point in time. Therefore, ongoing surveillance is needed to understand the seasonal changes to the athlete pain profile and NSAID use throughout the year. One obvious limitation of a cross-section study like this is the potential for recall bias, particularly for injury and surgical history and off-season NSAID use. It is also possible that athletes may have over or under reported their NSAID use. Also pain perception is subjective. However, use of questions from previously validated surveys for pain [33] and NSAID [4] use could limit the impact by being comparable to previous literature. Another limitation is that the response rate was 26% and most of the responding athletes were females, thus generalisability of our findings might be limited in male collegiate athlete populations. However, research into female athletes are limited; thus, these data help address the general lack of research into the female athlete. Only a small number of athletes participating in collision sports completed the survey, limiting generalisability of these results to these populations as pain profiles might be different. Further, most athletes included in this study were Caucasian, decreasing the generalisability of these findings to other ethnic groups. However, typically collegiate athletes are homogenous population, and therefore behaviours such as NSAID use and accessing these medications, and alcohol use might be similar. Finally, as only an online survey was used, results might be subject to single method bias. As pain and pain management is complex, mix methods and qualitative research are needed explore these complex multi-dimensional constructs.

## Conclusions

Half of female athletes and one in three male athletes reported pain. Back, knee and foot/ankle pain and knee, back and shoulder pain is common in female and male athletes respectively. Female athletes with current pain had greater odds of consuming NSAIDS and most of female and male athletes reported obtaining NSAIDs through self-purchase. These results suggest that collegiate athletes consume NSAIDs without medical supervision for pain, and there is a need to investigate and intervene on health literacy within this population, in order to potentially curtail harmful health consequences.

## Supplementary information

**Additional file 1: Appendix A**. Survey questions used to collect pain and non-steroidal anti-inflammatory drug use data

**Additional file 2: Appendix B**. Missing Data

## Data Availability

Data is available upon reasonable request to the authors.

## Abbreviations

NCAA: National Collegiate Athletic Association
NSAID: Non-Steroidal Anti-Inflammatory Drugs
OSTRC: Oslo Sports Traumatic Research Centre for Overuse Injury Questionnaire
95% CI: 95% Confidence Intervals
BMI: Body Mass Index
