# Supplementary material for: Epidemiological profile of pain and non-steroid anti-inflammatory drug use in collegiate athletes in the United States
Source: BMC Musculoskelet Disord. 2020 Aug 19;21:561. doi: 10.1186/s12891-020-03581-y (PMC7437034; doi:10.1186/s12891-020-03581-y)
Supplement: Supplementary file 1 — Additional file 1: Appendix A. Survey questions used to collect pain and non-steroidal anti-inflammatory drug use data [file 12891_2020_3581_MOESM1_ESM.docx]

Appendix A. Survey questions used to collect pain and non-steroidal anti-inflammatory drug use data

|  | Response |
| --- | --- |
| **Demographics** |  |
| Sex | Male, Female |
| Weight |  |
| Height |  |
| Please select the ethnicity that is most applicable to you | American Indian or Alaskan Native, Asian, Black or African American, Hispanic or Latino, Native Hawaiian or Other Pacific Islander, White, Do Not Wish to Answer |
| What year are you in college? | Freshmen, Sophomore, Junior, Senior, Graduate |
| What is your main varsity sport you currently play in college? | Basketball, Baseball, Swimming, Football, Soccer, Softball, Golf, Cross Country, Tennis, Lacrosse, Track and Field, Volleyball, Triathlon, Wrestling, Other |
| Approximately how many seasons have you played your main sport? | Number of seasons |
| **Pain** |  |
| To what extent have you experienced pain related to your sport during the last week?^5^ | (0–No pain; 8–Mild pain; 17–Moderate pain; 25–Severe pain; 25–Cannot participate at all*) |
| Please select all that apply and indicate which side(s) | Hip/Groin, Knee, Ankle/Foot, Spine (upper, mid, or low back), Shoulder, Elbow, Hand, Face |
| **Non-Steroidal Anti-Inflammatory Drug Use** |  |
| *Are you currently taking any Non-Steroidal Anti-Inflammatory Drug (NSAIDs; Common brand names: ibuprofen, Advil, Aleve, Motrin, Addaprin, Midol, Aspirin, Naprosyn, Toradol, Celebrex, and Vioxx) at this time for any reason for your sport?^7^*  If participants answered yes, participants were then asked the following series of questions:  (1) How often do you use NSAIDs while competing IN-SEASON?  (2) How often do you use NSAIDs while training in the OFF-SEASON?  (3) From whom do you PRIMARILY receive your NSAIDs?  4) What is the typical dose of NSAIDs you take at a given time for sports related activity? | Yes, No  Response for the follow up questions 1 and 2.  frequently (3-7 days per week), regularly/occasionally (1-2 days per week), infrequently (1-3 times per month) and never  Response for question 3.  self-purchased, parents, teammates/friends, coaches, athletic trainers, doctors, and other  Response for question 4.  2 pills, 4 pills 6 or more pills, 200mg, 400mg, 800mg, and higher than 800mg. |
| **Alcohol** |  |
| 1. Have you drank alcohol in the past 30 days?^30^ 2. How many occasions have you had alcohol in the past 30 days?^30^ | 1. Yes, No 2. 1 to 2, 3 to 5, 6 to 9, 10 to 19, 20 to 39, 40+ occasions |
| **Sleep** |  |
| On average, how many hours of sleep do you get per night?^27^ | Under 7, 7 to 8, more than 8 |
| **Surgical History** |  |
| Have you ever had orthopedic surgery (including bone, ligament, or joint surgery)?^4^ | Yes, No |
| **Injury History** |  |
| Have you ever had any sport-related injuries leading to more than 4 weeks of reduced participation in exercise, training, or sport?^4^ | Yes, No |

*Added as an extra option to original answer options
