# Supplementary material for: Epidemiological profile of pain and non-steroid anti-inflammatory drug use in collegiate athletes in the United States
Source: BMC Musculoskelet Disord. 2020 Aug 19;21:561. doi: 10.1186/s12891-020-03581-y (PMC7437034; doi:10.1186/s12891-020-03581-y)
Supplement: Supplementary file 2 — Additional file 2: Appendix B. Missing Data [file 12891_2020_3581_MOESM2_ESM.docx]

Appendix B. Missing Data

1. Heat Map


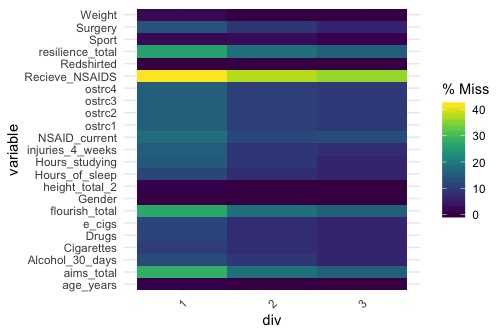


1. Analyses between those that answered and did not answer where they received non-steroid anti-inflammatory drugs.

| Variable | Did not answer  (n = 89) | Answered  (n = 141) | p-value |
| --- | --- | --- | --- |
| Age (years) | 19 (18-20) | 19 (18-20) | 0.516 |
| Mass (kg) | 67.8 (11.4) | 67.7 (12.9) | 0.994 |
| Height (m) | 1.70 (0.09) | 171 (0.08) | 0.129 |
| NCAA Division  Division 1  Division 2  Division 3 | 37 (42%)  29 (32%)  23 (26%) | 52 (37%)  48 (34%)  41 (29%) | 0.760 |
| Surgical History  Orthopaedic Surgery  No Surgery | 11 (17%)  55 (83%) | 25 (18%)  116 (82%) | 0.999 |
| Injury History  Injury  No Injury | 31 (49%)  32 (51%) | 78 (55%)  63 (45%) | 0.511 |
